# Silybin, a Major Bioactive Component of Milk Thistle (*Silybum marianum* L. Gaernt.)—Chemistry, Bioavailability, and Metabolism

**DOI:** 10.3390/molecules22111942

**Published:** 2017-11-10

**Authors:** Michal Bijak

**Affiliations:** Department of General Biochemistry, Faculty of Biology and Environmental Protection, University of Lodz, Pomorska 141/143, 90-236 Lodz, Poland; michal.bijak@biol.uni.lodz.pl; Tel./Fax: +48-42-635-4336

**Keywords:** silybin, silymarin, chemistry, bioavailability

## Abstract

Milk thistle (*Silybum marianum*) is a medicinal plant that has been used for thousands of years as a remedy for a variety of ailments. The main component of *S. marianum* fruit extract (silymarin) is a flavonolignan called silybin, which is not only the major silymarin element but is also the most active ingredient of this extract, which has been confirmed in various studies. This compound belongs to the flavonoid group known as flavonolignans. Silybin’s structure consists in two main units. The first is based on a taxifolin, the second a phenyllpropanoid unit, which in this case is conyferil alcohol. These two units are linked together into one structure by an oxeran ring. Since the 1970s, silybin has been regarded in official medicine as a substance with hepatoprotective properties. There is a large body of research that demonstrates silybin’s many other healthy properties, but there are still a lack of papers focused on its molecular structure, chemistry, metabolism, and novel form of administration. Therefore, the aim of this paper is a literature review presenting and systematizing our knowledge of the silybin molecule, with particular emphasis on its structure, chemistry, bioavailability, and metabolism.

## 1. Introduction

Milk thistle (*Silybum marianum* L. Gaernt.), sometimes called wild artichoke, is a medicinal plant that has been used for thousands of years as a remedy for a variety of ailments. [1]. The milk thistle is an annual to biannual plant of the *Asteraceae* family, flowering in July–August with reddish-purple flowers. Milk thistle needs to grow in a warm atmosphere and dry soil, and will grow up to 3 m high and 1 m across. However, it most commonly reaches 0.9–1.8 m in height. Native habitats of milk thistle are Southern Europe, Southern Russia, Asia Minor and Northern Africa, and is it naturalized in North and South America as well in South Australia [2]. Milk thistle flower heads are 4–8 cm in diameter and contain around 50–200 tubular florets (individual flowers forming part of a group of flowers), which have a 13–25 mm dimension with color ranging from magenta to purple. The bracts below the flowers are broad and rigid with a rounded appendage ending in a spine. This plant has one long taproot. Milk thistle has variegated dark and light green spiny leaves with a length up to 75 cm and width up to 30 cm that are smooth on the upper surface and hairy on the lower surface. The leaves have milky-white veins, which inspired its common name of *Silybum marianum.*

**Figure 1:**
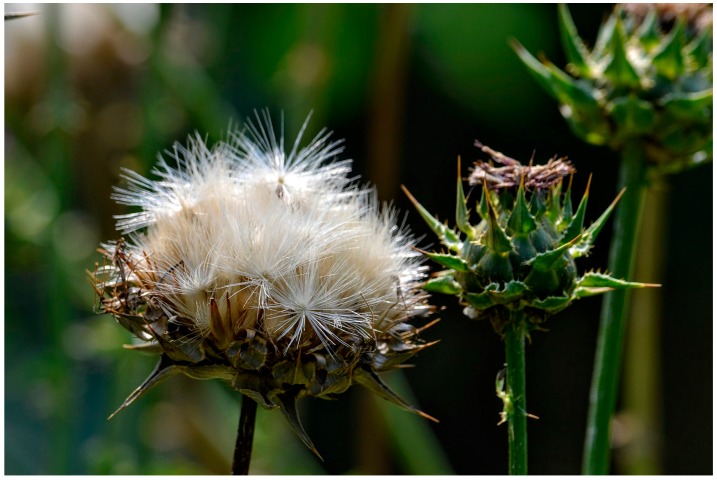
Milk thistle (*Silybum marianum* L. Gaernt.).

There is confusion about whether milk thistle has fruits or seeds. Botanically correct, this plant has a cypselae, which looks like a seed but is technically a fruit. Each fruit (having a cocoa-like odor and an oily, bitter taste) is about 5–8 mm long, up to 2–3 mm wide, and 1.5 mm thick, with a glossy, brownish-black to greyish husk. They are hairless but have a white, silky pappus (an appendage) of fine bristles. The fruits are joined together around the ring (Figure 1).

In *Silybum marianum* fruits have been used by mothers for stimulating milk production. *S. marianum* is also associated with a legend that the white veins of the plant’s leaves were caused by a drop of the milk of the mother of Jesus. When leaving Egypt with the infant Jesus, she finds a shelter in a bower formed from the thorny leaves of the milk thistle. Thanks to this legend, milk thistle is sometimes called Mary thistle, St. Mary’s thistle, holy thistle, blessed virgin thistle or Christ’s crown [2].

*Silybum marianum* has been used since the time of ancient physicians and herbalists to treat a range of liver dysfunctions and gallbladder disorders. The first records of this plant can be found in the Old Testament (Genesis 3:18). In ancient Greece, the *Silybum marianum* was administered to cure liver dysfunction. It has been also discovered that Indian and Chinese medicines used *Silybum marianum* in clinical practice for liver and gallbladder problems [2]. Its hepatoprotective action has been proven by many researchers [3,4,5]. Thanks to its healthful properties, silymarin—an extract of milk thistle fruits—was classified by the WHO in the 1970s as an official medicine with hepatoprotective properties [6].

Silymarin represents 1.5–3% of the fruit’s dry weight and is an isomeric mixture of unique flavonoid complexes—flavonolignans. The main representatives of this group presented in silymarin are silybin, isosilybin, silychristin, isosilychristin, silydianin, and silimonin [2,7,8,9,10,11]. The chemical composition of milk thistle fruit besides flavonolignans also include other flavonoids (such as taxifolin, quercetin, dihydrokaempferol, kaempferol, apigenin, naringin, eriodyctiol, and chrysoeriol), 5,7-dihydroxy chromone, dehydroconiferyl alcohol, fixed oil (60% linoleic acid; 30%, oleic acid; 9% palmitic acid), tocopherol, sterols (cholesterol, campesterol, stigmasterol, and sitosterol), sugars (arabinose, rhamnose, xylose, and glucose), and proteins [2]. However, the highest concentration, comprising approximately 50–70% of the extract, is silybin, which is the major bioactive component of extract, which has been confirmed in various studies. The silybin concentrations typically found in common pharmaceutical products containing a silymarin range of 20–40% [11]. Besides the hepatoprotective action, silybin has strong antioxidant properties and modulates a variety of cell-signaling pathways, resulting in the reduction of pro-inflammatory mediators [12]. Silybin is also studied as a potential anticancer and chemo-preventive agent [13]. Research performed last year demonstrates that silybin is able to inhibit serine proteases involved in the blood coagulation process [14,15], as well as reduce blood platelets’ response to physiological agonists [16,17,18,19].

## 2. Silybin Structure and Chemistry

The chemical structure of silybin was first established by Pelter and Hansel in 1968, by careful examination of ^1^H-NMR (100 MHz, DMSO-*d*_6_) and MS spectra [20]; however, the absolute silybin configuration in positions C-2 and C-3 was discovered using a degradative method by the same researchers in 1975 [21]. Silybin, which is also called flavobin, silliver, silybine, silymarin I, silybina, and silybine, has a molecular formula of C_25_H_22_O_10_ and a molecular weight of 482.441, CAS No. 22888-70-6 (data obtained from the pubchem website). The silybin structure consists in two main units. The first is based on a taxifolin, which is a flavononol group in flavonoids. The second is a phenyllpropanoid unit, which in this case is conyferil alcohol. These two units are linked together into one structure by an oxeran ring [22,23].

The silybin structure can be characterized as a small, highly functionalized molecule with alternating carbo- and hetero-cycles. This compound is very stable under Brønsted acidic conditions, while in the presence of Lewis acids or under basic conditions, the stability is reduced. Additionally, prolonged heating over 100 °C causes disruptions of its skeleton. Silybin is quite resistant to reduction, but is easily oxidized by two oxygen molecules to 2,3-dehydrosilybin [24].

Silybin in neutral aqueous solutions has weak acidic properties, with a p*K*_a_ of 6.63 for the 5-OH group, 7.7–7.95 for the 7-OH group, and 11.0 for the 20-OH group [25,26].

In silybin’s structure, we can recognize five hydroxyl groups, which are the primary targets of the derivatization process. Three of these hydroxyl groups (5-OH, 7-OH, and 20-OH) possess a phenolic nature. The 5-OH group has a very strong hydrogen bonding to the adjacent oxo group, which is in the conjugation with the aromatic ring and acts as a free electron pair donor to the hydrogen bond with the 5-OH group. The 7-OH and 20-OH have similar properties, although the C-7 OH group is more reactive than the 20-OH group due to its lower steric hindrance and the presence of a hydrogen bond. The C-23 OH group have properties leading to the esterization or the oxidation of carboxylic groups. The C-3 OH group can easily be oxidized (even with atmospheric oxygen) to a ketone, which is responsible for the creation of a 2,3-dehydrosilybin. Silybin is poorly soluble in polar protic solvents (EtOH and MeOH), and insoluble in non-polar solvents (chloroform and petroleum ether), but highly soluble in polar aprotic solvents such as DMSO, acetone, DMF, and THF [24].

**Figure 2:**
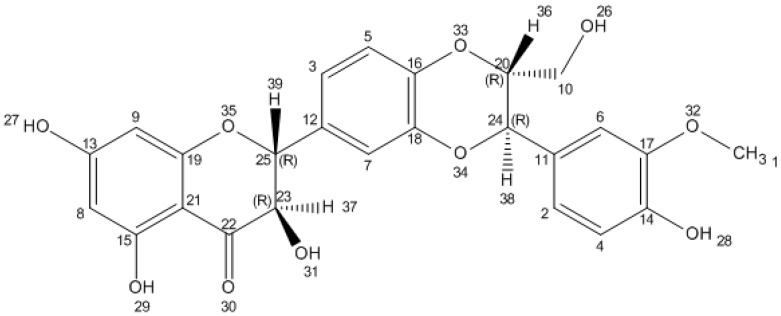
Chemical structure of silybin. Structure generated from InChI code from http://pubchem.ncbi.nlm.nih.gov/.

In nature, silybin occurs in the form of two *trans* diastereoisomers: A and B. These two diastereoisomers are differentiated with respect to reference positions C-10 and C-11 in the 1,4-benzodioxane ring [22,27]. Silybin A and silybin B both have 1H and 13C NMR spectra, which are very similar (without any characteristic signals), and impede the detailed identification of individual isomers [28]. The most popular method of separation of these two diastereoisomers is high-performance liquid chromatography (HPLC), which is able to differentiate the molecules by analysis of the retention time [29,30]. Despite problems with NMR spectra, the absolute configurations of these diastereoisomers have been established as follows: Silybin A is 2*R*, 3*R*, 10*R*, 11*R* isomer with a proper IUPAC name of (2*R*,3*R*)-2-[(2*R*,3*R*)-2,3-dihydro-3-(4-hydroxy-3-methoxyphenyl)-2-(hydroxymethyl)-1,4-benzodioxin-6-yl]-2,3-dihydro-3,5,7-trihydroxy-4*H*-1-benzopyran-4-one. Silybin B has a configuration of 2*R*, 3*R*, 10*S*, 11*S* and IUPAC name (2*R*,3*R*)-2-[(2*S*,3*S*)-2,3-dihydro-3-(4-hydroxy-3-methoxyphenyl)-2-(hydroxymethyl)-1,4-benzodioxin-6-yl]-2,3-dihydro-3,5,7-trihydroxy-4*H*-1-benzopyran-4-one (Figure 2) [28,31,32].

The silybin diastereoisomers also have different optical rotation parameters: silybin A has an ${\left[\alpha \right]}_{D}^{23}$ +20.0° (*c* 0.21, acetone), while silybin B has an ${\left[\alpha \right]}_{D}^{23}$ −1.07° (*c* 0.28, acetone) [32]. The differences between silybin A and silybin B are also observable after compound crystallization. Silybin A (MeOH–H_2_O) forms yellowish flat crystals with a melting point of 162–163 °C, while silybin B generates yellow grain crystals (MeOH–H_2_O) with a melting point of 158–160 °C [28].

In *Silybum marianum*, flavonolignans are formed by combination of flavonoid and lignan structures. This occurs through oxidative coupling processes between flavonoids and a phenylpropanoid. The first hypothesis of this process was described by Freudenberg [33] and confirmed by Hansel [34,35]. Silybin biosynthesis from (+)-taxifolin and coniferyl alcohol is an oxidative process, catalyzed by peroxidase enzyme [36]. The first step of this process involves a single electron oxidation of substrates, leading to the generation of free radicals, derived from both taxifolin and coniferyl alcohol. These individuals react in the O-β coupling stage, which results in the formation of an adduct. Subsequently, the adduct undergoes cyclization by the addition of the phenol nucleophile to the quinine methide, generated by the coniferyl alcohol. The formation of two silybin diastereoisomers indicates that the radical coupling reaction is not stereospecific [36,37].

## 3. Silybin Metabolism

After oral administration, silybin undergoes extensive enterohepatic circulation. The elimination half-life of silybin is approximately 6 h [38]. It has been established that 3–8% of orally administrated silybin is excreted in an unchanged form in the urine. About 80% of silybin is excreted as glucuronide and sulfate conjugates with bile (silybin concentration in bile is 60–100 times higher than in serum, and attains a level of even 0.1 mM) [38,39,40]. It is assumed that 20–40% of bile silybin is recovered, whereas the remaining part is excreted via feces [38]. Silybin undergoes both phase I and phase II of biotransformation in liver cells [41]. Studies conducted in the last decade very clearly show that silybin interacts with a limited number of cytochromes (CYP) [42]. Several in vitro studies have suggested that silymarin extracts and various individual constituents inhibit CYP450 2D6, CYP450 2E1, CYP450 3A4, CYP450 2C9, and CYP450 2C8 [43,44,45]. However, Kawaguchi-Suzuki et al. [46] demonstrated that silymarin does not have any significant influence on the activities of CYP450 1A2, CYP450 2C9, CYP450 2D6, or CYP 450 3A4/5.

Using nuclear magnetic resonance (NMR), Jancova et al. [47] demonstrated that silybin is metabolized in vitro by CYP450 2C8 into *O*-demethylatedsilybin (major), and both mono- and dihydroxy-silybin (minor) metabolites. The presence of these metabolites was also confirmed by Gunaratna and Zhang [48] using liquid chromatography-mass spectrometry (LC-MS). Additionally, they also identified a number of other hydroxylated metabolites of silybin, although the site of hydroxylation was not determined by NMR and the exact structure of these metabolites remains unknown. Even so, phase I plays a marginal role in silybin in vivo metabolism [49].

Silybin monoglucuronides and diglucuronides, as well as silybin monosulfates and silybin diglucuronides sulfate, are all formed during phase II of silybin’s biotransformation [41]. Experiments with ovine liver glucuronyl transferase described by Kren et al. [50] demonstrated that silybin is glucuronidated in three -OH groups (C-5, C-7, and C-20). But in humans, glucuronidation of silybin is mainly at C-20 and C-7. Additionally, a very important role in silybin’s metabolism is played by the stereo-selectivity of the glucuronidation process. Silybin B is glucuronidated more efficiently, and the glucuronidation is much preferred in the C-20 position. Silybin A is glucuronidated with a similar efficiency in both the C-7 and C-20 positions [31].

The silybin glucuronides formed in phase II are then transported by biliary flow to the intestinal tract, where bacterial enzymes cleave sugar moieties and release silybin aglycones, which can be absorbed again, thus promoting enterohepatic circulation [39,51].

## 4. Bioavailability and Pharmacokinetics in Different Forms of Silybin Administration

Due to its highly hydrophobic and non-ionizable chemical structure, silybin displays poor water solubility of less than 50 μg/mL, and this has a great influence on its bioavailability [52]. However, the solubility of silybin significantly increases in various organic solvents: for example, the solubility parameters of silybin in transcutol, ethanol, polysorbate 20, or glyceryl monooleate increase to 350.1, 225.2, 131.3, and 33.2 mg/mL, respectively [41]. After oral administration, silybin is rapidly absorbed in the stomach (with a Tmax of about 2–4 h and a t_1/2_ of about 6–8 h). However, as mentioned previously, the absorption efficiency is rather low [40,53,54]. Studies performed on rat models have shown that the absolute oral bioavailability of the pure form of silybin is at a level of 0.95% [55].

Silybin bioavailability in the gastrointestinal tract is dependent on various factors, such as the concentration of the preparation and the presence of additional substances with a solubilizing character (e.g., fat, proteins, amino acids, cholesterol, or other flavonoids) [56].

Silybin concentrations in blood after oral administration of conventional preparations based only on silymarin extract are considered to be low. One of the first studies of silybin bioavailability was made by Lorenz et al. [39]. In their study of six healthy subjects, the maximum serum concentrations (Cmax) after 240 mg of silybin administration were low and ranged from 0.18 to 0.62 µg/mL. However, the levels in secreted bile were approximately 100 times higher than in the serum, varying between 11 and 44 µg/mL. In a study by Usman et al. of healthy male volunteers who received an oral 200 mg dose of silymarin tablets, Cmax values were 1.9 ± 0.1 and 2.9 ± 0.3 µg/mL; the area under curve (AUC) parameters were 10.8 ± 0.4 and 11.2 ± 0.7 µg/mL × h; Tmax parameters were 1.8 and 1.9 h, while t_1/2_ parameters were 2.5 and 3.8 h [57]. A comparison of the oral bioavailability of three different silymarin preparations containing silybin with different levels of fragmentation—Liverman’s capsule, Legalon^®^ capsule, and silymarin tablets—on 24 healthy volunteers was made by Kim et al. [58]. Each subject received a silybin dose of 120 mg in a 3 × 3 crossover study. The results obtained demonstrated differences in the maximum plasma concentrations obtained for these preparations of 6.04, 1.33, and 1.13 µg/mL, respectively, and AUC parameters were 13.9, 5.59, and 4.24 µg/mL × h, respectively. These results show that silybin bioavailability is dependent on the form of silymarin administration.

Silybin bioavailability can be significantly improved by adding solubilizing substances to the standard silymarin pharmaceutical product [49]. Other potential ways of increasing silybin’s oral bioavailability is by using phosphatidylcholine [2,59] in its preparation, as this creates phytosomes. Phytosomes are known to be a phytolipid delivery system, forming a bridge between convectional delivery systems and novel delivery systems. Phytosomes are a complex between a natural product and natural phospholipids, such as soy phospholipids. This complex is obtained through the reaction of stoichiometric amounts of phospholipids and the substrate in an appropriate solvent [60,61]. One of the first pharmacokinetic studies of preparations using these kinds of complexes was made by Barzaghi et al. [62] using silipide (IdB 1016)—a complex of silybin and phosphatidylcholine. The results they obtained showed an evident increase of silybin bioavailability after oral administration in healthy humans. Cmax, after the administration of 120 mg of silybin equivalents, was 298 ng/mL for silipide and 102 ng/mL for normal silymarin, while the AUC values were 881 and 257 ng/mL × h, respectively. These values were very similar after single doses as well as after 8-day administration. The effect of increased bioavailability of a complex of silybin and phosphatidylcholine was probably related to passage of the drug through the gastrointestinal tract. The next comparative pharmacokinetics study of silipide and the pure form of silybin was conducted by Morazzoni et al. [63]. The Cmax levels of unconjugated and total silybin after silipide administration of a single oral dose (200 mg/kg as silybin) were 8.17 and 74.23 µg/mL, respectively, while mean AUC (0–6 h) values were 9.78 and 232.15 µg/mL × h, respectively. After administration of the native form of silybin, the plasma levels of both unconjugated and total compound were under the analytical detection limit. Li et al. [64] examined the pharmacokinetics of a silybin–phosphatidylcholine complex (also known as a phytosome) in healthy male Chinese volunteers. Plasma levels of silybin were determined in 20 subjects after administration of single oral doses of the silybin–phosphatidylcholine complex (equivalent to 280 mg of silybin). Silybin from this complex was rapidly absorbed from the gastrointestinal tract (Tmax ranged from 0.67 to 2.67 h, with a mean of 1.4 h. The Cmax value in plasma was 4.24 ± 2.30 µg/mL, and AUC reached a level of 5.95 ± 1.90 μg/mL × h. The study was performed on the dogs model, which also demonstrated enhanced silybin bioavailability in a phytosome complex of phosphatidylcholine and silybin. The Cmax, Tmax, and AUC (0–24 h) values for total plasma silybin were 1310 ± 880 ng/mL, 2.87 ± 2.23 h, and 11 200 ± 6520 ng/mL × h, respectively, while the same parameters for a standardized silymarin extract were 472 ± 383 ng/mL, 4.75 ± 2.82 h, and 3720 ± 4970 ng/mL × h [65].

Another formulation that was created to increase silybin bioavailability after oral administration is a compilation of silybin, phosphatidylcholine, and vitamin E, which considerably enhanced silybin’s solubility [66]. A study was performed on 12 healthy volunteers using this formulation, and showed that after oral intake of a complex of silybin, phosphatidylcholine, and vitamin E (corresponding to 47 mg of silybin), the bioavailability of silybin was much higher in comparison to typical silymarin granules containing 58 mg of silybin (plasma concentrations of 213 ng/mL vs. 18 ng/mL, respectively) [67].

The next proposition for enhancing silybin’s oral bioavailability is the formation of bile salts mixed micelles. Many authors [68,69,70,71,72,73] have postulated that bile salts are able to increase the oral bioavailability of poorly water-soluble drugs by enhancement of the lipophilic substance transport across biological membranes [74]. Yu et al. [52] prepared silybin–sodium cholate/phospholipid-mixed micelles (with a mean particle size of 75.9 ± 4.2 nm) and tested their bioavailability on dogs in doses of 90 mg of silybin equivalents. The largest solubility of silybin was found to be 10.0 ± 1.1 mg/mL in the optimum formulation of mixed micelles. Compared to silybin-*N*-methylglucamine, after oral administration the relative bioavailability of the mixed micelles was 252.0%. The *C*max values were 107.0 and 94.7 ng/mL, respectively, but the AUC values were 13,372.4 and 505.8 ng/mL × h. Tmax was similar for both preparations—1 h. A creation of silymarin-loaded liposomes containing bile salt (SM-Lip-SEDS) produced a 4.8-fold enhancement in the oral bioavailability of silybin in rats. Cmax and AUC values (after treatment of 20 mg/kg dose) for SM-Lip-SEDS were 1.296 ± 0.137 µg/mL and 18.406 ± 1.481 µg/mL × h, respectively, while for silymarin powder these values were 0.640 ± 0.132 µg/mL and 3.824 ± 0.355 µg/mL × h [75].

The next most promising way of enhancing silybin’s bioavailability is the Self-Micro Emulsifying Drug Delivery System (SMEDDS). This is a drug delivery system that uses a microemulsion achieved by chemical rather than mechanical means. Drugs coated in this way are well known for their potential as alternative strategies for delivery of hydrophobic drugs [76]. The formulation of SMEDDS is an isotropic mixture of drug, oil, emulsifier, and co-emulsifier. These substances form a fine oil-in-water (*o*/*w*) microemulsion under gentle agitation following dilution by aqueous phases, with a particle size of less than 100 nm [77,78,79,80]. This spontaneous formation of an emulsion in the gastrointestinal tract presents the drug in a solubilized form, and the small size of the droplets formed provides a large interfacial surface area for drug absorption [77]. Additionally, apart from solubilization, the existence of lipids in this formulation improves bioavailability by directly affecting drug absorption [76]. The study silybin administered to dogs by Yu et al. using SMEDDS was composed of ethyl linoleate, Cremophor EL, ethyl alcohol, and saline (single doses of silymarin 50 mg/kg), and showed that this formulation has about 2.2-times higher bioavailability than hard capsules. AUC (0–12 h) and Cmax were 4.75 ± 0.26 μg /mL × h and 1.85 ± 0.09 μg/mL for SMEDDS, and 2.09 ± 0.15 μg /m × h and 1.06 ± 0.04 μg/mL for the Liverman’s^®^ capsule and Legalon^®^, respectively. The relative bioavailability of the SMEDDS to the Legalon^®^ was 227%, although in the case of the Tmax parameter, no significant difference was observed (*p* > 0.05) for the two formulations. Additionally, in Yu et al.’s experiment, the silymarin was successfully solubilized 327-fold by SMEDDS (130.8 mg/mL), which was closer to that in polysorbate 20 [81]. Wu et al. [54] examined silybin bioavailability in a rabbit model (at doses of 300 mg/kg) using SMEDDS formulations composed of silymarin/ethyllinoleate/Tween 80/ethyl alcohol. Relative bioavailability of silybin administered as SMEDDS was radically increased at an average rate of 1.88- and 48.82-fold when compared to the silymarin PEG 400 solution and suspension, respectively. Maximum concentration obtained in plasma was 1.01 ± 0.21 µg/mL, while AUC was 6.23 ± 1.75 µg/mL × h. The use of SMEDDS (with particular size 67 nm) constructed from silymarin/glyceryl monooleate/polysorbate 20/HCO-50 resulted in an increased oral bioavailability of silybin. From the reference capsule—Legalon^®^. After its oral administration in rats (at dose 140 mg/kg), the bioavailability of the drug from the SMEDDS was a few times higher than the reference capsule: Cmax values were 24.79 ± 4.69 μg/mL and 3.47 ± 0.20 μg/mL, respectively, while AUC (0–6 h) were 81.88 ± 12.86 µg/mL × h and 22.75 ± 3.19 µg/mL × h, respectively. Additionally, Tmax for SMEDDS was 0.5 h, whereas for Legalon^®^ was 1.1 h [82].

One of the most effective methods of intensifying silybin bioavailability is creation of specific nanoemulsions and nanosuspensions. Silybin nanoemulsion can consist of sefsol-218 as an oil, Tween 80 as a surfactant and ethanol as a co-surfactant (having a nano-droplet size and low viscosity). Parveen’s results indicated that the stability of silymarin can be enhanced in nanoemulsion formulation using Tween 80 as a surfactant [83]. After oral administration of sefsol-218/tween 80/ethanol nanoemulsion to rats (20 mg/kg of silybin equivalents), the hepatoprotective effect was higher than for standard silymarin [84]. The other silybin nanosuspensions, containing 0.2% lecithin and 0.1%poloxamer 188 were capable of increasing drug transport across the Caco-2 cell monolayer. After oral administration of nanosuspension (20 mg/kg) in beagle dogs, this formulation significantly increased silybin bioavailability, when compared to the coarse powder (Cmax (μg/mL) 2.73 ± 0.30 vs. 1.53 ± 0.22 and AUC (µg/mL × h) 9552 vs. 3264, respectively) [85].

Another very interesting method of improving the dissolution and bioavailability of silymarin is use of solution-enhanced dispersion by supercritical fluids (SEDS). A study performed on rats orally administered 20 mg/kg silymarin as SEDS or silymarin powders showed that SEDS has much better bioavailability and pharmacokinetic parameters than silymarin powder: Cmax (μg/mL) 1.093 ± 0.249 vs. 0.57 ± 0.143, AUC (µg/mL × h) 5.017 ± 0.35 vs. 2.054 ± 0.074. Additionally SEDS, also increased t_1/2_ (h) 7.830 ± 3.204 vs. 2.938 ± 0.694 [75].

In one of the most promising studies performed in the last few years, surface-attached silymarin-loaded solid dispersion with an improved dissolution profile and enhanced oral bioavailability was formulated using silymarin, polyvinylpyrrolidone (PVP), and Tween 80. The drug solubility of the optimized solid dispersion prepared with silymarin/PVP/Tween 80 at a weight ratio of 5/2.5/2.5 increased by almost 650 times compared to standard silymarin powder. After oral administration in rats (equivalent to 140 mg/kg dose of silymarin), this formulation caused an increase in Cmax and AUC values (44.85 ± 11.42 μg/mL and 366.49 ± 93.62 µg/mL × h vs. 16.74 ± 1.63 μg/mL and 157.04 ± 36.29 µg/mL × h) [86].

## 5. Conclusions

In summary, silybin is a very interesting chemical compound, the use of which as a dietary supplement is increasing all around the world, and this explains the recent studies aimed at increasing its oral bioavailability.
